# Motor imagery ability scores are related to cortical activation during gait imagery

**DOI:** 10.1038/s41598-024-54966-1

**Published:** 2024-03-03

**Authors:** Martina Putzolu, Jessica Samogin, Gaia Bonassi, Carola Cosentino, Susanna Mezzarobba, Alessandro Botta, Laura Avanzino, Dante Mantini, Alessandro Vato, Elisa Pelosin

**Affiliations:** 1 Department of Experimental Medicine (DIMES), Section of Human Physiology, University of Genoa, Genoa, Italy; 2 Movement Control and Neuroplasticity Research Group, KU Leuven, 3001 Leuven, Belgium; 3 Department of Neuroscience, Rehabilitation, Ophthalmology, Genetics, Maternal, and Child Health, University of Genoa, 16132 Genoa, Italy; 4 IRCCS Ospedale Policlinico San Martino, Genoa, Italy; 5 Department of Biomedical Engineering, The Catholic University of America, Washington, DC USA; 6 National Center for Adaptive Neurotechnologies, Stratton VA Medical Center, Albany, NY USA; 7 College of Engineering and Applied Sciences, University at Albany – SUNY, Albany, NY USA

**Keywords:** Motor imagery, Gait, Motor imagery ability, Event-related desynchronization (ERD), Electroencephalography (EEG), Neurophysiology, Motor control, Sensorimotor processing

## Abstract

Motor imagery (MI) is the mental execution of actions without overt movements that depends on the ability to imagine. We explored whether this ability could be related to the cortical activity of the brain areas involved in the MI network. To this goal, brain activity was recorded using high-density electroencephalography in nineteen healthy adults while visually imagining walking on a straight path. We extracted Event-Related Desynchronizations (ERDs) in the θ, α, and β band, and we measured MI ability via (i) the Kinesthetic and Visual Imagery Questionnaire (KVIQ), (ii) the Vividness of Movement Imagery Questionnaire-2 (VMIQ), and (iii) the Imagery Ability (IA) score. We then used Pearson’s and Spearman’s coefficients to correlate MI ability scores and average ERD power (*avgERD*). Positive correlations were identified between VMIQ and *avgERD* of the middle cingulum in the β band and with *avgERD* of the left insula, right precentral area, and right middle occipital region in the θ band. Stronger activation of the MI network was related to better scores of MI ability evaluations, supporting the importance of testing MI ability during MI protocols. This result will help to understand MI mechanisms and develop personalized MI treatments for patients with neurological dysfunctions.

## Introduction

Motor imagery (MI) can be defined as a dynamic brain state during which representations of a given motor act are internally rehearsed in working memory without any overt motor output^1^. Over the past century, MI has been used to improve motor skills in athletes, musicians, and singers^2^ and promote motor learning processes and motor abilities in neurorehabilitation^3^. In addition, during the last two decades, with the increasing number of scientists involved in developing brain-computer interfaces (BCIs), motor imagery gained a spike of interest, being at the basis of many BCI-based prostheses or BCI systems designed to control external devices^4^.

So far, evidence supports the effectiveness of MI training in improving motor performances in healthy subjects, the elderly^5^, and individuals with neurological diseases^6^, even if to a lesser extent than motor practice^7^, in particular over time^8^.

How to explain the effect of MI on motor skill learning is the goal of several studies investigating the neurophysiological mechanisms and neuroplasticity processes at the basis of this mental practice. The working hypothesis confirmed by several investigations suggested that imagined and actual movements share brain substrates and activate similar motor representations^9–11^. Moreover, the degree of similarities and differences in brain activation patterns when comparing motor execution (ME) and MI highly depends on the type of imagery. Motor imagery can be performed by creating a visual representation of the action in the first- or third-person (i.e., visual imagery, VI) or a mental simulation associated with a kinesthetic feeling of a movement (i.e., kinesthetic imagery, KI). Soldokin and colleagues^12^ showed that VI and KI certainly shared physiological characteristics with motor execution but simultaneously presented significant differences that cannot be neglected. Their analyses of the functional connectivity of the activated network during the three experimental conditions (i.e., ME, VI, and KI) provided new insight into the neural mechanisms underlying these three motor behaviors. This work also highlighted the crucial role of the instructions given to the subjects before they perform the motor imagery task; in several experiments reported in the literature, the researchers don’t specify the modality of motor imagination (visual vs. kinesthetic) the subjects need to use, making the results difficult to interpret and generalize.

In this endeavor to explore the role of motor imagery in motor skill learning, it is worth mentioning that other studies hypothesized and demonstrated that the differences between motor practice and motor imagery can be partially explained by the lack of somatosensory input^7,8^ and movement inhibition in motor imagery^13^.

Nevertheless, the neurophysiological mechanisms at the basis of MI effectiveness are still not wholly understood, also due to the lack of accurate and objective tools to measure the imagery ability of each individual^14^. Since imagination can differ in every person^15,16^, researchers have developed methods for assessing the subject's mental capacity to rehearse a specific movement or action. Most of these techniques are based on psychological questionnaires where subjects are asked to score the imagery vividness from a first- or third-person perspective (Vividness of Movement Imagery Questionnare^17^, VMIQ) or, in addition, the intensity of the sensation generated by the imagined action (Kinesthetic and Visual Imagery Questionnaire^18^). Mental chronometry^19^ represents a more quantitative technique that measures the difference between the duration of the imagined movement and its physical execution; a slight difference between the two durations reveals a well-trained motor imagery ability.

Investigating the relationship between the scores of such psychological tests and the physiological measures of the brain activity recorded during different motor imagery tasks could help to identify which brain regions are more involved in determining the subject’s motor imagery ability. Guillot et al.^20^ used functional magnetic resonance imaging (fMRI) to identify the differences in brain activation between skilled and unskilled imagers during the execution and imagination of finger movements. With the equal objective, Lorey et al.^21^ used the same neuroimaging technique by performing a parametric analysis of how the perceived imagery vividness is associated with activating the parietal-premotor network when performing an MI of right-hand movements. Event-related potentials from the electroencephalogram (EEG) recorded over the sensorimotor cortex were analyzed by Toriyama and colleagues^22^ to correlate the vividness of kinesthetic motor imagery with alpha and beta band neural activity during voluntary contraction and motor imagery of wrist dorsiflexion. Unlike most MI studies involving hand gestures or upper limb movements, Van der Meulen and colleagues^23^ explored the neural correlates of a gait imagery task. First, they divided the subjects according to the obtained MI ability scores into two groups (i.e., the good and bad imagers). Then, they used functional images to analyze the differences in brain activation between the two groups during a kinesthetic and visual gait imagery task. They also performed supplemental parametric analysis to correlate the MI ability scores with the neural activity of the regions activated during gait imagery.

Our work aims to explore the correlation mentioned above by overcoming the limitation of classifying the subjects as good or bad imagers based on an arbitrary threshold as the median of the MI ability scores^23^. We analyzed the relationship between MI ability and brain activity recorded in nineteen healthy subjects during a gait MI task. We assessed the correlation between the subjects' scores obtained from MI ability tests and the electroencephalographic brain activity recorded during a gait motor imagery task. In this sub-analysis of our previous paper^24^, we focused our analysis of recorded high-density EEG signals (hdEEG) by measuring event-related desynchronization (ERD) in the theta (4 ÷ 8 Hz), alpha (8 ÷ 13 Hz), and beta frequency band (13 ÷ 30 Hz), applying a custom-developed pipeline for source localization. We hypothesized a correlation between the ability to imagine, measured using mental chronometry tests and questionnaires, and the amplitude of ERD in brain signals recorded from the cortical areas in the MI network.

## Methods

### Subjects

The data analyzed in this paper have been collected from 19 healthy adults, as described in a recent publication from our group^24^. We excluded from participation subjects (i) with previous experience with MI techniques or training, (ii) with a history of neurological diseases, or (iii) being treated with any medication that affected the central nervous system. We performed a sub-analysis after removing two subjects since the Imagery Ability Score was unavailable. The subjects provided informed written consent for participating in this study that was approved by the local ethical committee (CER Ref.1293 of September 12th, 2018) and conducted in conformity with the Declaration of Helsinki. All the included subjects declared to be right-handed and right-footed.

### Motor imagery ability assessment

We collected each participant’s response to the Kinesthetic and Visual Imagery Questionnaire^18^ (KVIQ) and the Vividness of Movement Imagery Questionnaire-2 (VMIQ)^17^ to assess the subjects' motor imagery ability.

The KVIQ measures***,*** on a five-point ordinal scale***,*** the ability of the subject to mentally represent ten movements performed with all body segments. Subjects are required to imagine each of the ten movements and rate the clarity and intensity of the imagined movement from the first-person perspective. The clarity of the image is evaluated using a visual sub-scale (KVIQ-v) from a score value equal to one***,*** meaning “no image,” to five, meaning "image as clear as seeing." The sensation intensity (KVIQ-k) is evaluated using a kinesthetic sub-scale from a score equal to one representing “no sensation” to five, meaning “as intense as executing the action.” The higher the score, the better the MI ability.

The VMIQ evaluates the imagery ability in three different conditions: internal (VMIQ-int) and external (VMIQ-ext) visual imagery and kinesthetic imagery (VMIQ-k)^25,26^. We chose this questionnaire because each of the twelve actions to be imagined involves lower-limb movements or is related to gait tasks. The score ranges from 12 to 60; the lower the score, the better the personal estimation of the motor imagery ability. The VMIQ-int and the VMIQ-ext questionnaires consider a score equal to one point as "perfectly and clearly, as normal vision" and a score equal to five as "no image at all." In the VMIQ-k questionnaire, a score of one means "perfectly and clearly, as the normal feel of movement," and a score of five points means "no feel at all".

Since we asked the participants to imagine themselves walking visually, we only used the scores from the VMIQ-int, VMIQ-ext, and the KVIQ-v to evaluate MI ability.

To include an objective measure in our study, we calculated the Imagery Ability (IA) score defined by Beauchet and colleagues^27^. The IA score measures the temporal differences between performed and mentally simulated movements by combining two mental chronometry tests^28^: the Timed Up and Go^29^ (TUG) and the 10 Meters Walking Test^30^ (10MWT).

First, for each test, a delta score (DS) was computed as follows:

$$DS= \frac{({T}_{real}-{T}_{imag})}{({T}_{real}+{T}_{imag})/2} \times 100$$

T_real_ is the time required to execute the task, and T_imag_ is the time needed for the mental simulation of the same task. Then, the IA score was calculated as the absolute value of the mean of the two delta scores^27^. The lower the IA score, the better the subject’s ability to imagine.

### Gait motor imagery task

**Figure 1:**
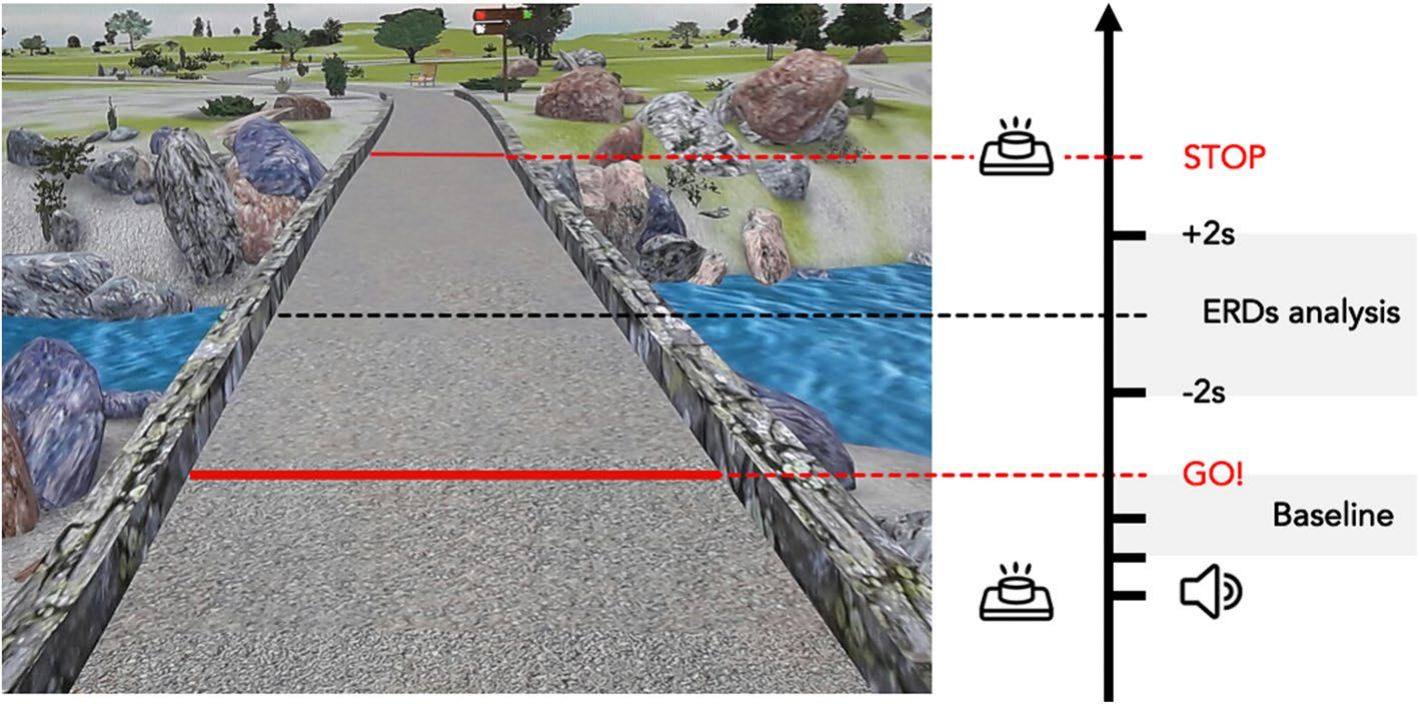
Motor imagery task. The picture shows a straight pathway. The black arrow indicates the task progression. The first red line (GO!) indicates the starting point of MI task; the second red line indicates the end of the MI task (STOP). The grey box "Baseline" indicates the time window selected as ERDs baseline analysis. The grey box "ERDs analysis" indicates the time window selected for ERDs analysis during MI task.

After the motor imagery ability assessment, the participants were seated in a quiet room in front of a screen displaying a picture representing a straight pathway with two red lines at the beginning and the end (Fig. 1). The task consists of visually imagining themselves (from outside) walking on a straight path, keeping their eyes open, starting from the first red line, and stopping when crossing the second one.

To start the MI task, the subjects had to press a push-button and wait for a GO signal presented on the screen, preceded by a 4-s audio countdown ("3, 2, 1, GO") before beginning to imagine. When they mentally crossed the second red line, they had to press the button again. We selected visual MI due to our interest in better understanding “higher level processes” implicated in gait control (e.g., navigation) and because, in real-life scenarios, visual MI is the most commonly employed strategy. The gait MI task consisted of three blocks of 10 trials each, totaling 30 trials. During the inter-trial period, a fixation cross was presented on the screen for three seconds to prevent mental fatigue. We implemented precautionary measures during our experiment to address potential confounds related to muscle contractions. Before starting, participants were given explicit instructions to avoid muscle activation, especially in the lower limb area. Throughout the experiment, researchers consistently reminded participants not to engage in muscular activity, ensuring adherence to MI tasks.

### EEG recording and processing

#### EEG data collection

We recorded the subjects' brain activity using a high-density EEG system equipped with 128 channels (Brain Products GmbH, Munich, Germany) with a sample rate of 1 kHz. We positioned the active wet EEG electrodes according to the 10–5 system^31^, and used the FCz electrode as a physical reference to increase the signal-to-noise ratio as recommended by the manufacturer. We used a trigger box connected to the hdEEG system to record external events as the push-button presses and to sync them with the brain signal. We recorded the vertical and horizontal electrooculographic signals (vEOG/hEOG) to identify and remove ocular-related artifacts from scalp registration. During each experimental session, we verified that the electrode impedance remained below 5kΩ throughout the session using the Brainvision Recorder software (Brain Products GmbH, Munich, Germany).

#### EEG data preprocessing

We first identified and corrected noisy channels by interpolating their time course from the adjacent channels. We fixed between 0 and 15 channels for each participant (median = 6; IQR = 6), mainly in the frontal area. Then, we used the EEGlab software tool^32^ to band-pass filter the signals (1 ÷ 80 Hz) and reject ocular and muscular artifacts embedded in the data using Independent Component Analysis (ICA)^33,34^. Each independent component (IC) was classified according to three parameters: the correlation of the power of the IC with the power of vEOG and hEOG signals; the coefficient of determination obtained by fitting the IC power spectrum with a 1/f function; the kurtosis of the IC time-course^35,36^. We used the parameters' thresholds as described in previous works^35,37^. The time courses of the ICs classified as bad were reconstructed at the channel level and subtracted from the hdEEG data. The average number of removed ICs in the datasets ranged between 5 and 35 ICs (median = 18.7; IQR = 15.4). Then we completed the cleaning step by applying, where necessary, a despiking on 0.2 s consecutive windows. Finally, we applied the average re-reference (AR) technique to re-reference the cleaned hdEEG recordings to a virtual reference calculated from all the available EEG recordings^38^. With such processing, no trials had to be discarded before proceeding to the following steps of source activity reconstruction and ERD analysis^39^.

#### EEG source localization

To localize the source of the EEG signals recorded from the scalp, we employed a robust and well-known automated pipeline^33,35,40,41^.

First, we built a head model based on a template magnetic resonance (MR) head image and template electrode positions ^35,41,42^ to calculate the leadfield matrix. The MRI head image was segmented into 12 compartments^41^, and the template electrode positions were rigidly co-registered to the head contour (i.e., the outer layer of the skin compartment). Subsequently, by using Simbio (https://www.mrt.uni-jena.de/simbio), the numerical approximation of the whole-head volume conduction model was calculated as a finite element model^43^. At last, we created the leadfield matrix that expresses the scalp potentials corresponding to each source configuration. More details about head modeling have been previously reported^24^.

Finally*,* to estimate the brain activity of each voxel within the source space, the artifact-free re-referenced scalp hdEEG and the realistic head model were used as input to the exact low-resolution brain electromagnetic tomography algorithm (eLORETA)^44^.

### Event-related desynchronization analysis

For the following analysis, we selected 32 regions of interest (ROIs), included in the AAL brain atlas^45^, and previously associated with MI of walking^23,46–58^ (Supplementary materials, Table S1). ROI coordinates were projected on the cortex of the template head. All voxels included in a spherical region with a 6 mm radius and centered in the ROI coordinates were used to calculate the ROI activity and defined as ROI masks. We used the principal component of these voxels' time courses to represent the ROI neural activity, and we analyzed these signals in the theta (4 ÷ 8 Hz), alpha (8 ÷ 13 Hz), and β frequency bands (13 ÷ 30 Hz).

The event-related desynchronizations (ERDs) were assessed using source reconstructed data. We used the Short-Time Fourier Transform to perform a time–frequency decomposition on each voxel time course by applying a moving Hamming window of 2 s, with 50% overlap between consecutive windows. Spectrograms were created in the frequency range 1 ÷ 80 Hz, at steps of 1 Hz, and epoched with a 4 s time window. In our ERD analysis, we considered as *MI task* the 4 s epoch (+ 2 s; -2 s) computed from the midpoint of each MI task and as *baseline* the 4 s epoch centered 2 s preceding the GO-signal in each trial.

The spectrogram epochs of MI tasks were averaged across trials, and the ERD intensity of each voxel was calculated as the percentage value of the relative difference between the epoch power at a given time point and the average baseline power for θ, α, and β band, separately^33^. Then, we created the ERD spatial maps by averaging the time–frequency values corresponding to the relevant frequencies within the same range. As the final step, we converted the ERD maps reconstructed in individual spaces into MNI space^35,41,42^.

### Statistical analysis

For each participant, ROI mask, and band, we selected the number of desynchronized (i.e., negative) grey matter voxels (i.e., dsv(i)) within the *i*th ROI mask in the individual desynchronization map. These values were normalized on the total number of voxels within the corresponding mask with the formula: $ds\%(i)=100\cdot \frac{dsv(i)}{\sum vox({mask}_{i})}$

All the results were corrected for age using a multiple regression method that removes the effect of age from the individual frequency-specific ERD maps. The individual mean amplitude of desynchronization (*avgERD)* within each ROI mask *(i)* was calculated as the average amplitude of the desynchronized voxels identified in the previous step for each band.

We used Spearman's rank correlation coefficient (⍴) to identify a possible relationship between the scores of visual imagery questionnaires (i.e., KVIQ-v, VMIQ-ext, and VMIQ-int) and the intensity of the desynchronization in each ROI of interest (i.e., *avgERD*) and the Pearson's correlation coefficient (R) for correlations between IA score and *avgERD*. Additionally, as a control analysis, we computed Spearman's rank correlation coefficient (⍴) to evaluate correlations between *avgERD* and the scores of kinesthetic imagery questionnaires (i.e., KVIQ-k, VMIQ-k). The alpha value was corrected for multiple comparisons^59^ and was set at 0.009. All analyses were conducted with MATLAB ® (R2018a, Math-Works, Natick, MA, USA).

## Results

Participants' demographic and MI ability test scores are summarized in Table 1. Data from seventeen volunteers, ten females, mean age 35.88 ± 13.36 (SD) years (range 20–49), were entered in the statistical analysis. The mean (± SD) imagery ability scores of the KVIQ-v, VMIQ-ext, and VMIQ-int were 43.41 ± 6.47, 23.82 ± 10.06, and 21.94 ± 9.27, respectively. The mean (± SD) delta score of TUG and 10MWT mental chronometry tests were of 0.17 ± 0.15 and 0.12 ± 0.08 s, respectively, determining a mean (± SD) IA score of 0.14 ± 0.08. The mean (± SD) kinesthetic imagery ability scores of the KVIQ and VMIQ, used as control analysis, were 41.00 ± 7.04 and 23.41 ± 8.48, respectively.

**Table 1:** Demographic characteristics and behavioral data.

Gender (n. female, %)	10 (58.82%)
Age (years)	35.88 ± 13.36
Education (years)	19 ± 2.21
IA (score)	0.14 ± 0.08
TUG (delta)	0.17 ± 0.15
10MWT (delta)	0.12 ± 0.08
KVIQ-v (score)	43.41 ± 6.47
KVIQ-k (score)	41.00 ± 7.04
VMIQ-ext (score)	23.82 ± 10.06
VMIQ-int (score)	21.94 ± 9.27
VMIQ-k (score)	23.41 ± 8.48

Values are presented as mean ± standard deviation.

*IA* imagery ability; *TUG* timed up and go test; *10MWT* 10 meters walking test; *KVIQ* kinesthetic and visual imagery questionnaire; *v*, visual; *k* kinesthetic; *VMIQ*, vividness of movement imagery questionnaire-2; *ext* external; *int* internal.

### Correlations

We found positive significant correlations between the VMIQ-ext score and the power of desynchronizations (*avgERD* data) of frontal areas, insula, and occipital regions in the theta band and of the cingulate cortex in the β band. All significant correlations indicated that a better VMIQ-ext score coincides with higher activation of areas involved in the MI network of gait.

**Figure 2:**
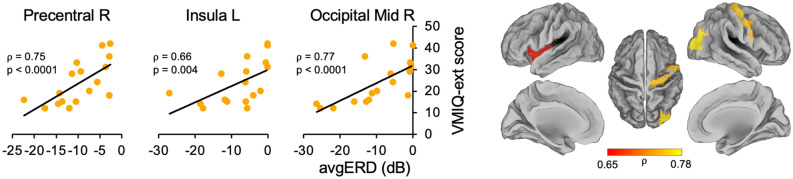
Correlations between VMIQ-ext score and *avgERD* during MI of gait in theta band. Significant correlations graphs between activations of the left insula, the right precentral area, and the right middle occipital region with VMIQ-ext score are shown on the left side of the picture. On the right side of the figure, magnitude of Spearman’s rank correlation coefficient (ρ) is visible in lateral, medial and dorsal views of brain maps. R = Right, L = Left, Mid = Middle, VMIQ-ext = Vividness of Movement Imagery Questionnaire-External, avgERD = mean amplitude of desynchronization.

**Figure 3:**
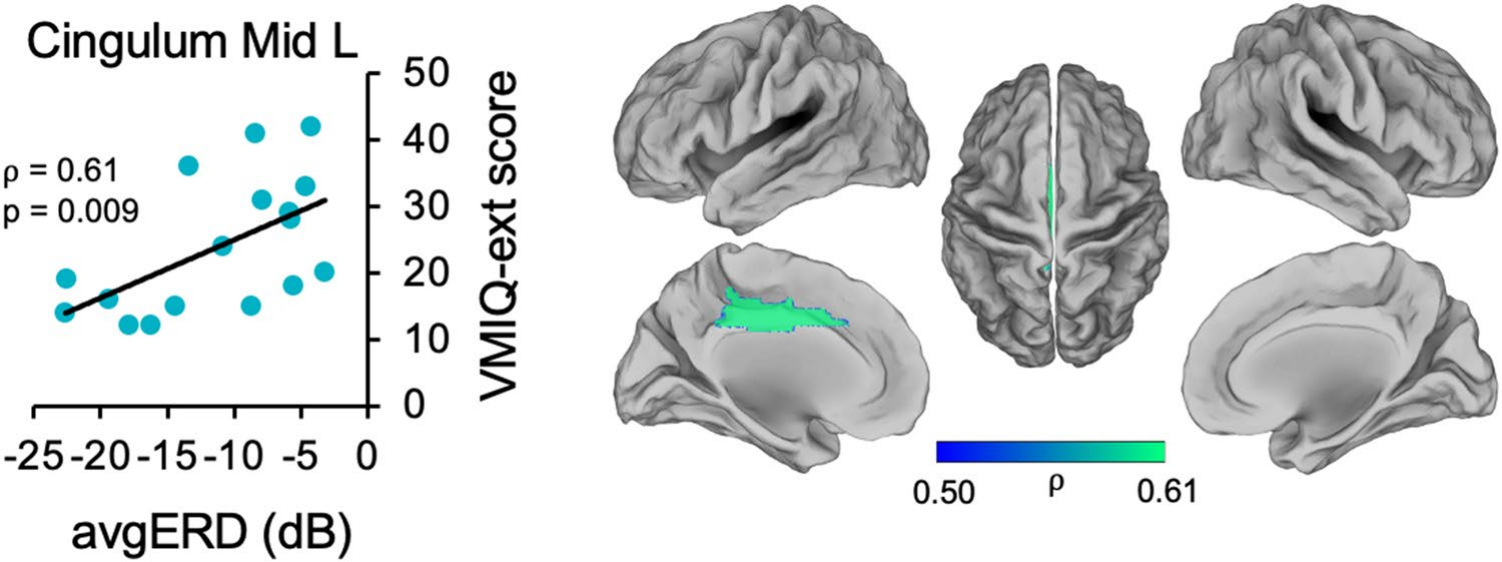
Correlations between VMIQ-ext score and *avgERD* during MI of gait in β band. The left side of the image displays significant correlation between the activations of the left middle cingulum with VMIQ-ext. On the right side of the figure, magnitude of Spearman's rank correlation coefficient (⍴) is shown in lateral, medial and dorsal views of brain maps. Mid = Middle, L = Left, VMIQ-ext = Vividness of Movement Imagery Questionnaire—External, avgERD = mean amplitude of desynchronization.

Precisely, in theta band, we found significant correlations between VMIQE and *avgERD* in the right precentral area (⍴ = 0.75, *p* < *0.0001*), in the left insula (⍴ = 0.66, *p* = 0.004), and in the right middle occipital region (⍴ = 0.77, *p* < *0.0001*). Moreover, a significant relationship was found with the VMIQ-ext score in the left middle cingulum (left: ⍴ = 0.61, *p* = 0.009) in the beta band. No significant relationships were found when correlating *avgERD* with KVIQ-v, VMIQ-int, and IA scores. Also, the control analysis, including correlations with VMIQ-k and KVIQ-k, did not reveal any significant results. Correlations between the power of desynchronization and MI ability score (*avgERD*-VMIQ-ext) are shown in Fig. 2 (θ band) and Fig. 3(β band). The left side of the figures displays scatter plots, whereas the right side shows the magnitude of Spearman's rank correlation coefficients in the lateral, medial, and dorsal views of brain maps.

## Discussion

In this study, we aimed to explore whether MI ability was associated with the cortical activity of brain regions of the MI network in a group of healthy subjects during a gait imagery task.

The main result of this study was that we found a significant relationship between the power of the activity in the areas already shown^37–50^ to be involved in MI of gait and the MI ability scores.

Precisely, a positive correlation was detected between VMIQ-ext scores and (i) ERDs θ band power of the right precentral area, the left insula, and the right middle occipital gyrus, and (ii) ERDs β band power of cingulate area.

Only a few studies have tried to investigate whether the individual ability to imagine vividly was associated with distinctive brain activity patterns^20,23,60,61^. The first paper, published in 1992 by Charlot and co-workers^60^, measured brain activity in healthy undergraduate volunteers, classified as "high" and "low" imagers, based on the score of two clinical tests (i.e., the Minnesota Paper Form Board and the Mental Rotations Test), during a visual imagery task consisting in a mental representation and exploration of an imaginary island. Using regional cerebral blood flow (CBF) imaging, they found that low imagers had a widespread CBF increase, whereas high imagers showed a more focal activation^60^. The authors explained this finding by hypothesizing a low cognitive function differentiation in bad imagers and, conversely, a more differentiated cognitive architecture in skilled imagers. Later, differences in brain activity, using functional magnetic resonance imaging, were investigated in participants showing high or poor MI ability during physical execution and mental imagery of finger movements^20^. Results revealed that good imagers had higher bilateral activation in the premotor parietal regions, known to have a crucial role in the MI network, with respect to bad imagers. By contrast, participants with poor MI ability manifested more significant posterior cingulate, orbitofrontal areas, and cerebellum activations, possibly reflecting a compensatory mechanism to counteract difficulties in creating a vivid representation of sequential movements.

Concerning evidence investigating differences among subjects with good and poor MI ability during MI of gait, it has been reported that imagery capacities influence functional brain activity even during the imagination of a simple and well-automatized motor task. Meulen and collaborators^23^ found that participants with good MI ability had higher cortical activation in the primary motor cortex, the prefrontal cortex, the thalamus, and the cerebellum with respect to those with lower imagery performance.

In line with these results, we also found that MI ability level influenced cortical recruitment, specifically in areas particularly involved in the MI neural network^23,46–58^. A positive correlation was found between the MI ability test scores and the right precentral area, suggesting that the better the IA, the more involvement in this area.

Moreover, scores obtained by volunteers during the third-person VMIQ test were significantly positively correlated with the *avgERD* of the left middle cingulum and left insula. These brain regions are recognized as part of the MI network, and their activity is crucial for performing MI, specifically when considering MI of usual gait^23,56,58^.

Finally, the analysis of theta rhythm revealed how the activity of visual areas during MI was significantly related to the VMIQ-ext score. Such correlation might be associated with the nature of our MI task (i.e., visual motor imagery). As previously mentioned, two types of MI do indeed exist: (i) kinesthetic MI, which involves sensations and perceptions of muscle contractions implicated in the imagined movement, thus being categorized as proprioceptive (or somatosensory) imagination, and (ii) visual MI, which requires visualization of the requested action, thus engaging areas within the visual network. In this study, we specifically focused on investigating visual imagery only due to its association with navigation and its prevalence as the most utilized strategy in real-world scenarios. More specifically, we identified a correlation between the activity of the right middle occipital region and motor imagery ability. We already demonstrated that in good imagers, the skill to image actions with the lower limb from an external perspective is associated with exploiting the brain areas related to the imagination of the visual environment and visuospatial navigation^24^ during a usual walking imagery task.

Notably, no significant correlations were found between brain activity and scores of the first-person visual perspective of KVIQ (i.e., KVIQ-v) and the VMIQ (i.e., VMIQ-int). This could be related to the nature of our gait imagery task, where the external strategy (i.e., see themselves walking on the street) might fit better when observing a path and imagining walking into that. A possible explanation might be represented by the different brain processes that occur when subjects have to execute visual MI in first-person respect to a third-person perspective. Indeed, it was recently speculated that first-person imagery uses a bottom-up strategy, thus taking into account actions and reactions to concrete aspects of the imagined environment. In contrast, third-person imagery uses a top-down strategy due to the integration of the MI event with its wider context, including experience of other events beyond the main one^62^.

It should be emphasized that none of the correlations remained significant after FDR correction when we considered desynchronization power and mental chronometry. This hints at the possibility that the IA score may not be the most effective measure for distinguishing between good and bad imagers, in contrast to findings reported in previous research^23^. However, the absence of statistically significant results could potentially be ascribed to performing the test in a single attempt without allowing the participants, who were engaging in a motor imagery task for the first time, to undergo any familiarization.

Further, no results revealed a negative correlation between MI ability tests and MI network activity. This supports the hypothesis that the finest MI ability is associated with higher recruitment of regions involved in the MI network.

Finally, no significant correlations emerged with the analysis of alpha activity. This lack of correlations could potentially be attributed to our approach of examining the entire α band (i.e., 8 ÷ 13 Hz) without distinguishing between its two components (i.e., low and high alpha). Indeed, it is worth noting that previous research suggests that the low component (i.e., 8 ÷ 10 Hz) is associated with the attentional demand of a task and in motor preparation. In contrast, the high component (i.e., 11 ÷ 13 Hz) reflects movement topographic organization and task-specific aspects^63^.

Several limitations of the study deserve attention. First, the small sample size lessened the strength of our results. Second, leg muscle activity was not recorded during the hdEEG registration. Nonetheless, previous studies showed that EMG activity of distal leg muscles recorded during seated position decreased. At the same time, during standing gait, MI tasks led to a facilitatory effect on proximal lower limb muscle activity^64,65^. According to these findings, we might suppose an irrelevant effect of leg muscle activity on EEG data acquisition. However, EMG recordings would have provided valuable insights into the potential relationship between muscle contractions, vivid imagery experiences, and event-related desynchronization patterns observed in EEG data. Third, even though cerebellar activity has been linked to the MI cortical network, data consistency of hdEEG in detecting signals from the cerebellum is still up for debate^24^. Fourth, all participants self-reported as being right-handed and right-footed. However, we did not verify this information by applying tests and questionnaires designed to assess handedness and footedness, such as the Edinburgh Handedness Inventory and The Chapman & Chapman Foot Dominance Scale^66,67^.

This work represents a step toward a more profound knowledge of the underlying mechanisms of motor imagery processes for implementing its use in the clinical setting and developing personalized training protocols based on the subject’s abilities. The next step toward this goal would be integrating the MI ability assessment, the neuroimaging of gait imagery, and actual walking in the same experimental framework.

Previous experiments confirmed that exploring gait mechanisms and dynamics can help understand and improve motor deficits in walking. Among them, several neuroimaging studies exploring brain dynamics during actual walking offer a valuable comparison with the results obtained from gait imagery tasks. Recent advances in artifact removal techniques for signal processing allowed the recording of EEG signals during treadmill walking associated with auditory signals as pacing cue tones^68^, visual signals as interactive Virtual Environment^69^, and robot gait orthoses to assist during the gait training^70–72^. These works uncovered significant characteristics of brain oscillations that would have been impossible to detect with the subject restricted in a lying position as during an fMRI recording session. This motion artifact-free recording technique has also been employed to compare the cortical mechanisms involved in bicycling with walking to understand why such ability is preserved in Parkinsonian patients with freezing of gait^73^. These studies represent a valuable framework for designing future experiments to explore the real effectiveness of MI training in improving rehabilitation techniques for people with movement disorders as a consequence of stroke, spinal cord injury, or Parkinson’s disease.

## Conclusions

In this study, we investigated whether MI ability could be associated with neural activity during a gait imagery task performed by healthy subjects during hdEEG registration. Our findings confirmed that scores obtained in the MI ability tests were related to the activations in the MI network, supporting the importance of testing MI ability in subjects involved in research and clinical protocols. This will help, first, in profoundly understanding the neural mechanisms underpinning MI and, second, in developing tailored physiotherapy protocols based on the IA of patients. Nevertheless, we know that MI is a complex task, and several other aspects should be considered besides subjective questionnaires and chronometry performance to measure MI ability in healthy subjects better. Hence, future studies are needed to confirm our findings and to elucidate whether the relationships between MI ability and cortical activations could be influenced both by participants' previous experience and the type of motor task (e.g., tasks based on subjects' motor repertoire and more complex tasks, such as dual-task gait).

## Data and code statement

Data supporting these findings are available from the corresponding author upon reasonable request.

### Supplementary Information

Supplementary Information.
