# Supplementary material for: Motor imagery ability scores are related to cortical activation during gait imagery
Source: Sci Rep. 2024 Mar 3;14:5207. doi: 10.1038/s41598-024-54966-1 (PMC10909887; doi:10.1038/s41598-024-54966-1)
Supplement: Supplementary file 1 — Supplementary Information. [file 41598_2024_54966_MOESM1_ESM.docx]

**Table 1s.** Masks selected from AAL atlas, regions they belong, and corresponding MNI coordinates

| **Brain region** | **Cluster/Area** | **Side** | **X** | **Y** | **Z** |
| --- | --- | --- | --- | --- | --- |
| Frontal area | Superior Frontal gyrus | L | −18 | 35 | 42 |
|  |  | R | 22 | 31 | 44 |
|  | Middle Frontal gyrus | L | −33 | 33 | 35 |
|  |  | R | 38 | 33 | 34 |
|  | Inferior Frontal gyrus_Opercular part  Inferior Frontal gyrus_Triangular part  Inferior Frontal gyrus_Orbital part | L | −48 | 13 | 19 |
|  |  | R | 50 | 15 | 21 |
|  |  | L | −46 | 30 | 14 |
|  |  | R | 50 | 30 | 14 |
|  |  | L | −36 | 31 | −12 |
|  |  | R | 41 | 32 | −12 |
|  | Medial Frontal gyrus_Superior  Medial Frontal gyrus_Orbital | L | −5 | 49 | 31 |
|  |  | R | 9 | 51 | 30 |
|  |  | L | −5 | 54 | −7 |
|  |  | R | 8 | 52 | −7 |
|  | Supplementary Motor area | L | −5 | 5 | 61 |
|  |  | R | 9 | 0 | 62 |
|  | Precentral gyrus | L | −39 | −6 | 51 |
|  |  | R | 41 | −8 | 52 |
| Parietal area | Postcentral gyrus | L | −42 | −23 | 49 |
|  |  | R | 41 | −25 | 53 |
|  | Superior Parietal gyrus | L | −23 | −60 | 59 |
|  |  | R | 26 | −59 | 62 |
|  | Inferior Parietal gyrus | L | −43 | −46 | 47 |
|  |  | R | 46 | −46 | 50 |
|  | Supramarginal gyrus | L | −56 | −34 | 30 |
|  |  | R | 58 | −32 | 34 |
|  | Precuneus | L | −7 | −56 | 48 |
|  |  | R | 10 | −56 | 44 |
| Temporo-Occipital areas | Superior Temporal gyrus | L | −53 | −21 | 7 |
|  |  | R | 58 | −22 | 7 |
|  | Middle Occipital gyrus | L | −32 | −81 | 16 |
|  |  | R | 37 | −80 | 19 |
| Cingulate cortex | Anterior Cingulate gyrus | L | −4 | 35 | 14 |
|  |  | R | 8 | 37 | 16 |
|  | Middle Cingulate gyrus | L | −5 | −15 | 42 |
|  |  | R | 8 | −9 | 40 |
|  | Posterior Cingulate gyrus | L | −5 | −43 | 25 |
|  |  | R | 7 | −42 | 22 |
| Insula | Insula | L | −35 | 7 | 3 |
|  |  | R | 39 | 6 | 2 |
| L = left, R = right | | | | | |
